# A simple and accurate rule-based modeling framework for simulation of autocrine/paracrine stimulation of glioblastoma cell motility and proliferation by L1CAM in 2-D culture

**DOI:** 10.1186/s12918-017-0516-z

**Published:** 2017-12-11

**Authors:** Justin Caccavale, David Fiumara, Michael Stapf, Liedeke Sweitzer, Hannah J. Anderson, Jonathan Gorky, Prasad Dhurjati, Deni S. Galileo

**Affiliations:** 1 Department of Chemical and Biomolecular Engineering, University of Delaware, Newark, DE 19716 USA; 2 Department of Mathematical Sciences, University of Delaware, Newark, DE 19716 USA; 3 Department of Biological Sciences, University of Delaware, Newark, DE 19716 USA; 4 Helen F. Graham Cancer Center and Research Institute, Christiana Care Health System, Newark, DE USA; 5 Department of Pathology, Anatomy, and Cell Biology, Thomas Jefferson University, Philadelphia, PA USA

**Keywords:** Rule-based model, Glioblastoma, Cell motility, Scratch assay, L1CAM, NetLogo2-D, Cell culture, Cancer, Biological modeling framework

## Abstract

**Background:**

Glioblastoma multiforme (GBM) is a devastating brain cancer for which there is no known cure. Its malignancy is due to rapid cell division along with high motility and invasiveness of cells into the brain tissue. Simple 2-dimensional laboratory assays (e.g., a scratch assay) commonly are used to measure the effects of various experimental perturbations, such as treatment with chemical inhibitors. Several mathematical models have been developed to aid the understanding of the motile behavior and proliferation of GBM cells. However, many are mathematically complicated, look at multiple interdependent phenomena, and/or use modeling software not freely available to the research community. These attributes make the adoption of models and simulations of even simple 2-dimensional cell behavior an uncommon practice by cancer cell biologists.

**Results:**

Herein, we developed an accurate, yet simple, rule-based modeling framework to describe the in vitro behavior of GBM cells that are stimulated by the L1CAM protein using freely available NetLogo software. In our model L1CAM is released by cells to act through two cell surface receptors and a point of signaling convergence to increase cell motility and proliferation. A simple graphical interface is provided so that changes can be made easily to several parameters controlling cell behavior, and behavior of the cells is viewed both pictorially and with dedicated graphs. We fully describe the hierarchical rule-based modeling framework, show simulation results under several settings, describe the accuracy compared to experimental data, and discuss the potential usefulness for predicting future experimental outcomes and for use as a teaching tool for cell biology students.

**Conclusions:**

It is concluded that this simple modeling framework and its simulations accurately reflect much of the GBM cell motility behavior observed experimentally in vitro in the laboratory. Our framework can be modified easily to suit the needs of investigators interested in other similar intrinsic or extrinsic stimuli that influence cancer or other cell behavior. This modeling framework of a commonly used experimental motility assay (scratch assay) should be useful to both researchers of cell motility and students in a cell biology teaching laboratory.

**Electronic supplementary material:**

The online version of this article (10.1186/s12918-017-0516-z) contains supplementary material, which is available to authorized users.

## Background

Glioblastoma multiforme (glioblastoma, GBM) is the most common and deadly form of brain cancer, with a patient survival time of about fourteen months with treatment [4] and an average adult (40+ years old) five year survival rate of 3.7% [11]. The current treatment for GBM involves surgical resection (removal), followed by treatment with radiation and drugs (e.g., temozolomide) targeting remaining tumor cells. However, GBM cells aggressively invade surrounding brain tissue from the start, so that cells invariably are left behind after surgical resection. These cells also are resistant to adjuvant chemo- or radiation-therapy, so that they initiate tumor regrowth. The recurring tumors often are even more aggressive in their spread, resulting in the extremely poor prognosis typical of GBM. We have shown that the neural adhesion molecule L1CAM (L1) stimulates GBM cell motility and invasion both in vitro and in vivo using a variety of approaches [1, 10, 17]. If mechanisms controlling GBM cell motility and invasion can be modeled and simulated, then this likely will aid in experimental design and predicting outcomes of experimental manipulations.

L1 affects GBM cell motility through autocrine/paracrine stimulation of integrin and fibroblast growth factor receptor (FGFR) signaling that appears to converge through focal adhesion kinase (FAK) [1, 10, 17]. Our in vitro experimental cell motility paradigm primarily has been the *SuperScratch* assay whereby an area in a confluent monolayer of cells is wiped or “scratched” clean with a pipet tip to leave a free edge within the confluent monolayer from which cells can migrate into the denuded area (see [1, 5]). We then collect sequential images of the scratch edge over time and subsequently measure motility rates of the individual cells over that time period, thus giving highly quantitative data on individual and collective cell motility. We have used multiple experimental treatments to elucidate L1 autocrine/paracrine stimulation mechanisms, including attenuation of L1 expression in L1-positive cells, ectopic expression of L1 in L1-negative cells, blocking L1 with specific antibodies and peptides, overexpression of a dominant negative form of FGFR, and blocking cell signaling using small molecule inhibitors of integrins, FGFR, and FAK in L1-positive vs. L1-negative cells [1, 10, 16, 17]. Based on our experiments so far, we theorize that transmembrane L1 is proteolyzed and released as a large ectodomain fragment from cells at the scratch edge to interact with the cells’ integrin and FGFRs to initiate cell signaling cascades that converge through FAK to stimulate cell motility and proliferation. This scenario has multiple variables, but is simple enough to be modeled based on several rules. We sought to determine if our observed experimental motility and proliferation behavior of GBM cells could be modeled accurately by using a set of simple rules. Also, such a model might be useful for predicting the outcomes of experiments that have not yet been performed.

The modeling framework described here is based in the NetLogo modeling environment and includes release of a stimulatory protein fragment (L1 ectodomain) from cells, integrin and FGFR receptor signaling pathways, and a downstream convergent FAK signaling pathway. This model is based on experiments done in the Galileo laboratory showing that human T98G GBM cells express membrane L1 when confluent, which acts to adhere neighboring cells, but cleave L1 at the scratch edge. The cleaved L1 ectodomain stimulates GBM cell motility through integrins and FGFRs that share a common downstream effector (FAK). This adhesive component can be turned off in the model for cells that do not exhibit this characteristic, and inputs are provided to control the degree of proliferation, the average cell velocity, inhibition of individual receptors, and several other parameters. Several hierarchical rules govern the motile and proliferative behavior of cells over a set time course (e.g., 24 h). We have found this model to accurately simulate the experimentally observed behavior of GBM cell lines in vitro to a surprising degree.

### Biological problem/context

We have chosen T98G human glioblastoma cells as the cells to be modeled and the widely used “scratch” or “wound” assay as the experimental paradigm. We have used these cells and this assay in multiple reports of GBM cell behavior and molecular mechanisms controlling it in vitro (see references above). T98G cells when confluent express the adhesion protein L1CAM (L1) as a transmembrane protein that homophilically binds adjacent cells together. At the scratch edge where cells are not completely surrounded by other cells, an unknown mechanism upregulates ADAM10 protease expression in these cells, which cleaves L1 to release the L1 ectodomain (L1ecto) fragment. This serves 2 functions in T98G. One function is to release the predominant L1-mediated intercellular adhesive bonds, and the other function is to allow L1ecto to bind to several integrin and FGF receptors to actively stimulate motility and proliferation. We previously found that when cells maintain intercellular adhesion via non-cleaved L1 and release L1ecto concurrently, the adhesive interactions predominate and inhibit any increase in motility [3]. Some GBM cell lines may constitutively cleave L1 and release L1ecto regardless of their state of confluency, so a predominant adhesive role for L1 would not apply. Thus, we have allowed for multiple adhesion scenarios in our model.

Our model is based on a dozen assumptions that are consistent with our experimental findings using established human GBM cell lines. These assumptions were based on qualitative and quantitative analysis of data (velocity measurements and movies) from our time-lapse motility experiments of T98G cell motility. The assumptions are:

1. There is no significant cell death in these cultured cell lines.
2. All cells in the population express L1 constitutively.
3. All cells are assumed to be the same size and shape (defined as “perfectcell” by NetLogo).
4. Behind the scratch edge, cells can move around somewhat, but are confined in the monolayer by crowding and do not pile up on top of each other.
5. Some confluent cells (e.g., T98G) also are held together by cell surface L1 serving as an intercellular adhesion molecule. In such a case, cell-cell adhesion via L1 is a predominant factor controlling monolayer confluency.
6. T98G cells at the scratch edge cleave L1 and release the L1 ectodomain (L1ecto) fragment.
7. Released L1ecto diffuses away from the cells into the media for potential paracrine signaling, however, cells releasing L1ecto are fully stimulated by their own autocrine L1ecto and do not respond to a chemotactic gradient.
8. A high local concentration of L1ecto exists around cells releasing it such that it binds to and saturates cell receptors to cause autocrine/paracrine stimulation.
9. Cells at the scratch edge are released from any intercellular adhesion (e.g., for T98G) and are positively stimulated to migrate by L1ecto.
10. L1ecto binding to integrin and/or FGF receptors initiates signaling cascades that converge at FAK to stimulate cell proliferation and motility.
11. Migrating cells initially are directed away from the confluent monolayer into areas of less crowding (i.e., scratch area), but develop more random directionality once they progress into an uncrowded area with open space on all sides.
12. Cells that migrate away from the scratch edge continue to release L1ecto and undergo continuous autocrine/paracrine stimulation.

## Methods

### NetLogo modeling

In our model, we created an environment that simulates a scratch, or *SuperScratch*, assay using NetLogo 6.0, which is a free multi-agent programmable modeling environment developed at Northwestern University’s Center for Connected Learning and Computer-Based Modeling (https://ccl.northwestern.edu/netlogo/). Our simulations in this environment show how T98G GBM cells release and interact with L1CAM ectodomain (L1ecto), which initiates signals to the cells in an autocrine/paracrine matter to affect cell proliferation and motility. The language used by NetLogo is simple and straightforward, which is beneficial when computer science background is limited. In this agent-based model in NetLogo, cells are the primary agent in the simulation and rules governing their behavior were written to produce recognizable behavior within the model. The interface allows user control over a variety of parameters. By using data collected over a range of experimental conditions (e.g., [1]) one can adjust the parameters in the model to produce simulated behavior similar to the experimental behavior observed in the laboratory.

**Fig. 1:**
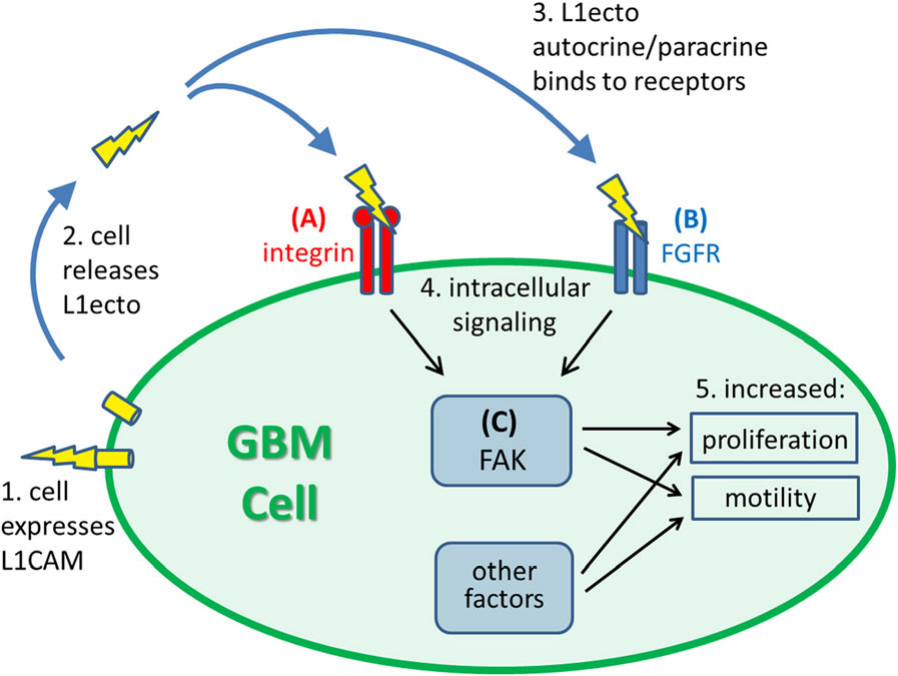
Connectivity map of the signaling pathways leading to motility and proliferation in a GBM cell. A GBM cell is shown as the large green oval. 1. GBM cell expresses L1CAM cell surface adhesion protein. 2. Cell releases the L1CAM ectodomain (L1ecto) after proteolysis from the cell surface. 3. L1ecto autocrine/paracrine binds to integrin (**a**) and FGFR (**b**) receptors to initiate intracellular signaling pathways (4.), which converge via FAK signaling (**c**). 5. Resulting cellular behavior is manifested as increased cell proliferation and motility

L1ecto binds to FGFR and integrin receptors immediately upon being shed, so the model was built under the assumption of binary signaling through both pathways leading to motility and proliferation. A connectivity map (Fig. 1) was developed to show how the signaling pathways lead to motility and proliferation in the GBM cell. Agents representing the GBM cells were created with an L1 ectodomain and internal pathways through the integrin receptor (A) and fibroblast growth-factor receptor, or FGFR (B). Both pathways are hypothesized to converge at the focal adhesion kinase (FAK) signaling pathway (C). This, amongst other factors and pathways, leads to observed motility and proliferation in the cell. We chose only to model this pathway and assumed other pathways contributed a constant rate of motility (base motility) and proliferation and had no dynamic effects on mitosis. However, this could be changed in the code by others.

The aforementioned 12 assumptions were translated into several rules in NetLogo that each cell would follow in a hierarchical manner:

1. The confluent cells behind the scratch edge are confined by the limited space between neighboring cells. With “cellular-adhesion” turned off, they wiggle because of their constant decisions (every tick) to look for, and move into, an area of more space.
2. When “cellular-adhesion” is turned on, it creates links between neighboring cells that act as adjustable springs holding them together. This is patterned after the “layout-spring” function.
3. When cells cross the scratch line by displacement or migration, they start releasing L1ecto ligand and begin migrating individually with a preference towards open space.
4. If open space surrounds a cell equally in all directions with a radius of “√2 * mu * dt” (mu being the motility, dt adjusting for time behavior), then the cell moves in a random direction.
5. If there is no open space surrounding a migrating cell, then the cell becomes stationary until space becomes available.
6. Decisions of how and where to move are made independently for each cell for each tick.

Sliders were made for the cell movement randomness, L1 ligand randomness, timescale, scratch line, and to enable inhibition of each pathway by a specified percentage. Cells that are at the scratch edge at the start of the simulation are colored green, as these are the cells that are tracked experimentally and in the simulation. This provides visual confirmation of the cell migratory pathways, which in general are towards open space (rarely, cells are seen traveling backwards both in our simulation and in experiments). All other cells at the beginning of the simulation are colored blue because they are behind the scratch edge and do not produce L1. As the simulation progresses, the green cells move away and the blue cells are exposed to the scratch edge. If cells progress past the scratch edge, they become red and begin releasing L1, providing a visual check for which cells should move faster due to lower adhesion and stimulation by L1.

NetLogo’s interface is set up with a number of features to allow easy control over the system. Controls are in blue-green windows and are described in Table 1. Monitors are tan windows and are described in Table 2. “Setup” calls the function “setup”, which clears the screen of the prior simulation and calls the function “initialize” so the next simulation can be prepared. “Run” is similar to a play button and will start or stop the simulation. It continuously calls the “go” function which contains the logic behind the simulation’s timing by controlling “ticks”. Ticks are NetLogo’s internal timer, and each agent in the model undergoes a singular random behavior each tick. We have chosen 1 tick in the model to represent 1 min of time.

**Table 1:** Explanation of Controls (Blue-Green Windows)

Control Parameter	Explanation	Functions Affected in code
Cellular-adhesion	Switch that turns on adhesion feature that tethers cells together with adjustable spring-like connections. Cells can wiggle or shift around in the monolayer to varying degrees.	update-params
time-scale	Slider that chooses the simulated runtime, currently ranging from 1 to 72 h. However, if a cell reaches the end of the simulation (towards the right) it will end prematurely.	go
randomness	Slider that determines the randomness of cell movements. The closer this is to 1, the cell path will be more random. When closer to 0, the cells move towards open space if possible, which usually results in straighter forward (east) movement.	cell-diffuse
L-randomness	Slider that determines the randomness of the yellow L1 ligand molecule movement. Closer to 1, L1 molecules have a better chance at random movement, vs. diffusing towards cells when set near 0.	L-diffuse
show-L?	Switch that allows the yellow L1 molecules to be hidden.	go
trailers?	Switch that will create colored trail markers behind the green cells that were initially at the scratch edge.	update-params
%_in_S-phase_Base	Input by the user to reflect the % of cells in the S-phase under normal, uninhibited conditions.	initialize
%_in_S-phase_Max_Inhibition	Input by the user to reflect the % of cells in the S-phase if all pathways were fully inhibited.	initialize
scratch-line	Slider that determines the initial cell density of the simulation and the location of the simulated scratch. The range is from −32 to 32, reflecting the x-coordinates of the NetLogo grid.	initialize; populate; make-scratch; L-production
LigandSpeed	Input from the user that has been determined to produce visual behavior that represents the speed of the ligand.	initialize
doubling-time	User input, in hours, to specify the average doubling time of the simulated cells.	populate; update-params; update-phase
Base_Motility_A	User input, in microns/ min, of the motility rate when the B pathway has been inhibited fully.	populate; update-params
Base_Motility_B	User input, in microns/ min, of the motility rate when the A pathway has been inhibited fully.	populate; update-params
Base_Motility_C	User input, in microns/ min, of the motility rate when the C pathway has been inhibited fully	Populate; update-params
UninhibitedMotility	User input, in microns/ min, of the motility rate for an uninhibited cell.	initialize
%DecreaseA	These sliders are set based on experimental data. They reflect the % inhibition in motility if the particular pathway is totally inhibited.	populate; update-params
%DecreaseB		
%DecreaseC		
A-inhibition	These sliders are set to reflect the degree to which that particular pathway is inhibited by a specific inhibitor.	initialize; populate; update-params
B-inhibition		
C-inhibition		
deviation-from-avg	This is an arbitrary input that is used to tune the variance in motility to that observed in experiments.	initialize
clear-cells	This button allows for the cells to be removed from the model. Typically, this option would be used to see the trailers more clearly.	
show-cells	This button will show the cells in the model after they have been cleared with the “clear-cells” button.	
show-scratch?	This switch gives the option to show the scratch line. The line itself is shown by the patches located to the left of the scratch line, which are colored white when the switch is on.	make-scratch
motility-check?	This switch is used to prevent the motility from being a negative number.	populate; update-params
S-phase_to_G1?	This switch is used to direct the change in S-Phase cells to G1 (on) or G2 (off).	update-params

**Table 2:** Explanation of Monitors (Tan Windows)

Monitor	Explanation
Cell Count	Gives the sum total of the cells in the simulation at any given time.
Avg. Motility	Displays the average motility rate, in microns/ min, of the green cells (initially at the edge).
Time (hours)	Shows the time elapsed in the simulation in hours.
theo. % S	Displays the variable “%S” so the user can see that the target number of cells in the S-phase throughout the simulation can be previewed based on varying the inhibition parameters.
% in S-phase	Displays the current % of cells in the S-phase during the simulation.
Ligand Count	Shows the total number of yellow ligand molecules (e.g., L1) in the simulation.
Cleaved Cells	Shows the total number of cells that have crossed the scratch line.
Uncleaved Cells	Shows the total number of cells that have not crossed the scratch line.
Phase Histogram (graph)	Illustrates the count of cells in each phase of the cell cycle with a histogram.
Average Motilty (graph)	Graphs the average motility of the green cells (initially at the scratch edge) throughout the simulation.
Cell Count (graph)	Displays the counts of the various cells throughout the simulation.
Phase Cycle (graph)	Graphs the percentage of cells in each phases of the cell cycle throughout the simulation.

Our simulation is set to be able to run for up to 72 simulated hours. This can be adjusted by right-clicking the “time-scale” slider, clicking “Edit…”, and adjusting the maximum value. However, if any cell manages to reach the far right edge of the environment, it will end the simulation at that point.

The “initialize” function sets up the simulation by performing the following jobs:

1. It resets the tick counter to zero.
2. The initial cell-density is determined based on the scratch-line position. Based on the size of the NetLogo grid underlying the simulation, a cell count is calculated based on how much room is behind the scratch-line. The entire area of the initial assay size is calculated based on the scratch-line so that the initial density can be obtained.
3. It also sets “dt” – a variable that was determined to produce recognizable behavior in the distances traveled by the cells upon each tick. This variable, multiplied by the square root of two, linearly translates the motility of the cells into a distance in the simulation that is reflective of actual cell behavior.
4. For proliferation simulation, an average response called “avg-resp” is calculated based on the percentage each pathway is inhibited as specified by the user at the beginning of the simulation. This is used to scale the cell cycle to reflect the level of inhibition.
5. Change in proliferation rates were determined by examining the proliferation data at the extremes and interpolating the data in between extremes and control situations. The “initialize” function next calculates a variable called “%S”, which calculates the theoretical percentage of cells in the S-phase for the next simulation. This can be seen on the monitor “theo. %S”.
6. Ligand parameters such as “mu-L”, which represents the speed of the ligand in micrometers per minute, is set according to the input from the interface labeled “LigandSpeed”.
7. The variable controlling the proliferation of cells, “prolif-factor” is the reciprocal of the average responses through the cellular signaling pathway. This is so that the cell cycle lasts longer when under inhibition, preventing mitosis. This will be explained in further detail below.

Controls are shown in blue-green windows, and are named after variables in the code. These can be adjusted before and during the simulation. These are listed in Table 1. Tan windows are monitors that display data during the simulation. Charts can be exported to a spreadsheet after the simulation is complete by right clicking the window and choosing “export.” These are listed in Table 2.

### Initial conditions

Anderson and Galileo [1] used small molecule inhibitors of integrins, FGFRs, and FAK to inhibit L1-mediated motility and proliferation on T98G and U-118 MG glioblastoma cell lines. Results suggested that intracellular FAK might serve as a convergence point between signals initiated by the FGFR and integrin receptors. The model uses the A pathway to represent the integrin receptor and the B pathway to represent the FGFR. The C pathway represents FAK and takes the maximum response between these two pathways and this translates directly into the motility rates generated in the model. We assume that L1ecto immediately binds to the external cell receptors upon being shed.

Each cell has an internal counter to keep track of their individual cell cycle. After entering the doubling time and data on the S-phase of the user’s cells into the interface, the model calculates a range across the doubling time to reflect each phase of the cell cycle. Each cell initially starts at a random point in the cell cycle. On the last tick of the cycle, the cell undergoes mitosis and creates two daughter cells. The daughter cells then begin their individually timed cell cycles to divide at the end of the specified length of the cycle.

The model also allows the user to view data on the S-phase under normal conditions of no inhibition and under inhibition of one or more signaling pathways. Our model assumes that proliferation changes linearly with inhibition (which may not always be biologically accurate). By allowing the user to specify the percentages of cells in the S-phase under no inhibition and under maximum inhibition, the model creates a line using a point-slope method. As the user changes the inhibition sliders, the phase boundaries - used within the model to separate phases on each cell’s cycle – are shifted to reflect the change in inhibition. The change in the S-Phase correspondingly will affect either the G1 or G2 phase, depending on whether the S-phase_to_G1? switch is on. If it is ‘on’, the change in the S-Phase will be accounted for in the G1 phase, rather than the G2 phase if the switch is turned to ‘off’.

### The Interface

Visual-aspects of the simulation can be found on the Interface tab. This houses the environment where agents, or cells in this case, interact. Pressing “Setup” spawns a constrained number of cells onto the screen in random places based upon the input parameters. Input parameters that are to be manipulated by the user are also located on the Interface. Cells are color coded based on their status in the simulation as described in Table 3.

**Table 3:** Key to Different Cell Colors

Cell Color	Description
Blue cells	Blue cells are those initially behind the scratch line, and reflect cells still under adhesion from L1.
Green cells	Green cells are those that are initially at the scratch line, and reflect cells that begin the simulation with L1 being cleaved and released and allow the cell to interact with the cleaved L1 to stimulate motility.
Red cells	Red cells are those that were blue, but have passed the scratch line because the cellular density surrounding this particular cell allowed them to do so (i.e., move to the right because cells initially at the scratch line moved to the right into open space).
Periwinkle, lime, and orange cells	Cells that are created through mitosis during the simulation will be the color of the parent but slightly lighter (e.g., division of a blue cell results in one daughter cell remaining blue, while the other is a lighter periwinkle color).

In order to change aspects of the simulation, the blue-green boxes on the Interface can be changed as desired. The switch, labeled “cellular-adhesion”, is better left off initially. By switching “cellular-adhesion” on, the simulation time increases dramatically unless the scratch edge is moved significantly to the left (e.g., about 10% of the way to the right). This is due to the increased number of calculations necessary for every cell behind the scratch edge, which can be minimized by moving the scratch line towards the left to result in fewer cells behind the scratch in the monolayer. This has no effect on the cells at the scratch line or ones moving freely beyond the scratch line. Thus, this is a minor limitation of the simulation because the cells of most interest (red, orange, and green) are unaffected. The cellular adhesion feature is useful with cell lines like T98G for visualizing that they are held together behind the scratch line by adhesive bonds, and that those bonds are broken as cells migrate into open space.

Experiments that would run for 24 h can be simulated in under five minutes (when “cellular-adhesion” is switched off). The time scale for the simulated experiment can be changed with the slider labeled “time-scale”, which allows adjustment of the simulated experiment from 1 to 72 h. This window was chosen based on run-time limitations, but can be adjusted by right-clicking the slider and choosing “Edit…” as described above.

The interaction of L1 with GBM cells was assumed to follow the hypothesized pathway described above. So that the model could easily translate to other research scenarios, the pathways were renamed so that they were not specific to the GBM-L1 interaction. The key to our connectivity pathway is as follows:

- Receptor A mirrors the integrin receptor and its pathway.
- Receptor B mirrors the FGFR receptor and its pathway.
- Receptor C mirrors the FAK receptor and its pathway of convergence.

Each pathway can be inhibited based on percentage. These are selected with the sliders located on the bottom of the interface labeled “A-inhibition”, “B-inhibition”, and “C-inhibition”, respectively. The user should note that the inhibition sliders directly affect both the motility and proliferation rates of the cells. Exact effects are discussed later.

The number of cells spawned in each simulation is dependent upon where the user sets the theoretical scratch line. When this line is set using the slider below the environment labeled “scratch-line”, NetLogo calculates an appropriate number of cells to fill the virtual culture dish behind the line with a near confluent layer of cells. The total number of cells can be found in a tan window on the left side of the screen, which updates continuously throughout the simulation as more cells are born. The concentration of cells has been pre-determined in the code in order to reflect a nearly confluent cell monolayer, but this can be altered for systems that may require a different cell density.

The left-hand side of the Interface also shows more information and allows more parameter manipulation. These all pertain to the parameters unique to our experiment, such as base motility values. “LigandSpeed” allows one to adjust how fast the ligand moves in the simulation. This speed is not reflective of a true measurement, but is selected arbitrarily in order to create a visual representation of ligand movement. Our LigandSpeed is set at 0.25, which allows for L1 to diffuse but remain primarily concentrated around cells that are interacting with the ligand.

Cell doubling-time can be based upon the type of cell being studied. Many cultured cell lines, including GBM cells, are observed to double about once a day, which we have reflected in our simulation. Again, this can be adjusted by changing the “doubling-time” parameter located on the interface.

Windows labeled “Base_Motility_A”, “Base_Motility_B”, and “Base_Motility_C” allow the user to set a baseline value for movement speed (in microns per minute) through pathways A, B, & C, respectively. Experimental data [1] showed uninhibited movement through solely the A (integrin) pathway (with B inhibited) to be about 0.15 μm per minute, and through the B (FGFR) pathway (with A inhibited) to be about 0.13 μm per minute. Note that Base_Motility input values must be set lower than the UninhibitedMotility value. Also, Base_Motility values for pathway A or B must be set greater than or equal to the value set for pathway C.

Next, two input windows labeled “%_in_S-phase_Base” and “%_in_S-phase_Max_Inhibition” allow the user to customize the percentage of cells in the S-phase. Experimental data from Anderson and Galileo [1] showed uninhibited T98G cells are 28% in S-phase, which drops to 8% under maximum inhibition. For the user with this data readily available, this is a small but influential part of the simulation that allows for more realistic trials, since proliferation can also be lowered. However, we have added only one input of lowered S-phase and not separate ones for each inhibition pathway.

On the bottom of the interface, a number of tan windows are found beneath the input windows, where information that updates throughout the simulation can be visualized. “Average Motility” tracks the mean speed of the green cells in the simulation, so it can be seen how fast the initially scratched cells move throughout the experiment, as done in our cell tracking experiments using MetaMorph software. All this data can be exported to a spreadsheet upon completion of a simulation by right clicking the desired window, choosing “Export…” and then saving the given *.csv* file.

“Theoretical % in S phase” displays the calculated percentage of cells that should be in the S phase at a given time equal to the user’s input parameters. This will match the “%_in_S-phase_Base” under no inhibition, and will match “%_in_S-phase_Max_Inhibition” when under max inhibition.

Next, “% in S-phase” displays the actual percentage of all cells in the S-phase at the current time step. “Uncleaved Cells” reports the number of blue and periwinkle cells behind the scratch line, while “Cleaved Cells” reports the number of red and green cells that traveled beyond the scratch line, including daughter cells. Finally, “Ligand Count” shows the number of yellow L1 molecules shown in the modeling world at the current time step.

Underneath the simulation, the average motility of the green cells is plotted over time. Next to this graph, there is a graph of the cell counts for each cell population over time. The legend shows the colors of each cell population. A phase histogram lets the user see the total number of cells in each phase of the cell cycle at any given moment. Cell cycle data also is displayed on the right as the relative percentages of the total cell population in each phase of the cell cycle over time (“Phase Cycle”).

A “deviation-from-average” input allows the average motility value of the green cells, as plotted on the histogram, to deviate over time, which occurs in experimentally collected data. A value of around 0.3 gives a graphical deviation similar to that observed experimentally. However, if this value is set too large, especially under inhibition, the average motility values can go negative because of the way they are calculated. In this case, the baseline will shift up along the Y-axis to denote where zero occurs. A “motility-check?” switch will prevent motility from going negative. It can be left on, with one exception: if the pathways are fully inhibited (i.e., if the motility is low) and the “deviation-from-avg” is greater than about 0.5, the Average Motility is much larger than it should be, which can cause the cells to not follow their typical behavior. If the switch is turned off, however, the cells follow their expected behavior, although motility can be negative. In most biological cases, this should not be an issue since the deviation from average is not usually that large. To visualize the most accurate cell velocity under all conditions, one may wish to keep the “motility-check?” switch off and just ignore any few negative peaks.

### NetLogo info

Under the Info tab, one can find standard NetLogo documentation provided by the creator of the model. Headings include What is it?, How it works, How to use it, Things to notice, Things to try, Extending the model, NetLogo features, and Related models. Descriptions have been entered under these headings for this model. They will be useful when this paper is unavailable to the user.

### The code

The first blocks of code define what the simulation is working with. We define agent breeds, called “turtles” in NetLogo, to be cells and ligands (Ls). The breed “trailers” is designated for the trailing lines that follow the movement of the cells. Next, we define what the cells and ligands own – their personal parameters. Below is a more detailed description of the internal parameters.

Cells:

- A, B, C
  - ○These are all calculated based on the base response by the cell to each individual pathway (found through experimental data) and the inhibition percentage specified by the user at the Interface.
- Cycle
  - ○This is a number between 0 and the doubling time of the cell, as specified by the user at the Interface. At each time step, “cycle” is incremented by one. A threshold for each phase of the cell cycle is calculated based upon the doubling-time and user specifications about S phase percentage. Upon reaching a new phase, “phase” is updated accordingly.
- Phase
  - ○Here is the name of the cell’s current phase in the cell cycle. This is updated based on the aforementioned “cycle” parameter. If “phase” is equal to mitosis or the M phase, a daughter cell is spawned.
- Mu
  - ○The speed of the cell in microns per minute. This is chosen to be the minimum rate of motility through the A, B, or C pathways.
- Released?
  - ○This is a Boolean (true or false) value that tells us if the cell has cleaved L1 or not. If “released?” is true, the cell will be red or green. Otherwise, it will be blue.
- trailerColor
  - ○This parameter is randomly generated at the beginning so that all the initial cells have a different trailer color.

The grid in the simulated world creates patches, which hold information regarding the concentration of cells and concentration of ligand on each patch. These are used in calculations based on cellular and L1 density.

Globals are variables that are used throughout calculations and are not simulated concretely in the environment. Most are self-explanatory, such as cell-count and initial-density, and are typically used for values that are reported in the Interface.

The first function is called when the user presses the “Setup” button. This is under “to setup”. This function clears the world of its current inhabitants, and sets the shape for the upcoming simulation. It then makes a call to the function “initialize”.

“Initialize” readies the world for the next simulation. It resets the tick count, which is NetLogo’s internal timer. For this simulation, 60 ticks are equal to 1 h. “Initialize” handles many of the front-end functions of the simulation, as described more detailed as follows:

- The cell count is calculated through the size of the world and the location of the scratch line.
- Cellular response to each pathway is recorded based upon the user’s initial inputs.
- The threshold for cell-cycle phase transitions, as specified earlier, is calculated next. This accounts for the user’s input parameters such as S phase percentages and inhibition.
- Finally, initialize calls “populate” and “make-scratch”
  - ○ “populate”
    - Creates the number of cells specified by the calculation in “initialize”
    - Looks for open space to place cells
    - Sets initial internal cellular parameters
  - ○“make-scratch”
    - Checks for cells on the scratch line
    - Changes color to green and Boolean “released?” to true
    - Creates visual line for scratch if “show-scratch?” switch is turned on

“Go” is the function called when the “Run” button is pressed on the Interface. The arrows on the “Run” button tell us that it continually calls the “go” function on each tick. This function handles most of the work performed during the simulation. Upon each tick, “go” does the following:

- Counting parameters are updated
  - ○ If all the cells have inexplicably died, the simulation halts
- Patches are told to update their density through a function call to “update-density”
  - ○ This function counts the number of cells and ligands on each patch and updates their concentration.
- Cells are told to do three things:
  - ○ “update-params”
    - ▪ This function checks if the cell has released L1 or not. Motility and phase are updated based upon the cell’s location, neighboring densities of cells and L1, and whether or not they have released L1 or not.
  - ○ “cell-diffuse”
    - Based upon a random number generated, the cell either takes a random step in a random direction, or it moves towards areas of lower cellular density.
  - ○ “L-production”
    - Here we decide if the cell will release a new L1 molecule to the world. The cell will do so if it has been released from adhesion (“released?” equals true).
    - The first check is if the cell’s position is past the scratch line. Then, it ensures that there are less than two cells in the path ahead of the cell. If there are less than two cells, there is a 5% chance of the cell creating up to four new L1 molecules.
- Next, ligands are told to move based on the specified LigandSpeed.
- Finally, it checks to see if the number of ticks has reached the end of the user’s specified time-scale. If it has, the simulation halts.

## Results

Although our particular model was developed using data from the Galileo laboratory, the agent-based modeling framework was created with parameters that could be tuned to reflect data collected in another laboratory or during hypothetical situations. This will allow users to:

1. Examine other cell systems that may use a similar pathway by adjusting parameters.
2. Manipulate the model to reflect in vitro scenarios after further research.
3. Simulate planned experiments to analyze results against a baseline experimental scenario.

**Fig. 2:**
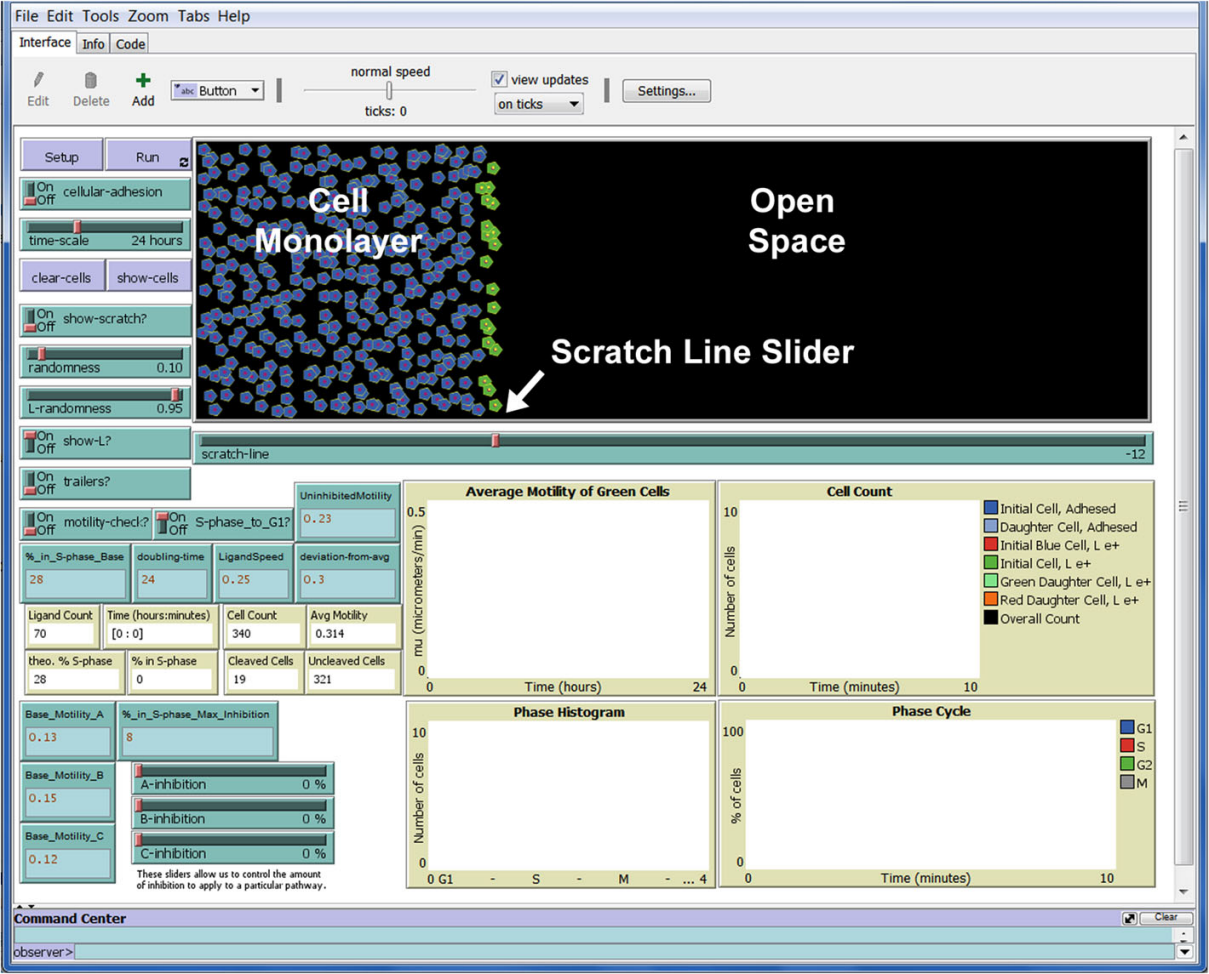
Initial arrangement of cells. The entire NetLogo graphic interface is shown. The cells at the scratch line are represented by the green cells at the edge of the cell monolayer of blue cells. The slider control for the position of the scratch edge is indicated. A white scratch line also can be switched on to denote where the green cells were located initially as the simulation proceeds. Cells will proceed to migrate into open space. Input controls are shown in the blue-green boxes

Several simulations are shown below at initial set up and end endpoints of cell motility. The simulations replicate the data collected in the Galileo laboratory (e.g., [1]) to a surprising degree. Fig. 2 shows cells initially ordered along the scratch edge before any motility has been simulated, as seen in the main simulation window. This is achieved by pressing the Setup button. The green cells are those along the scratch edge and are the ones that will be tracked for motility and proliferation. The blue cells are behind the scratch edge and do not get tracked, except as a component of the Cell Count graph.

**Fig. 3:**
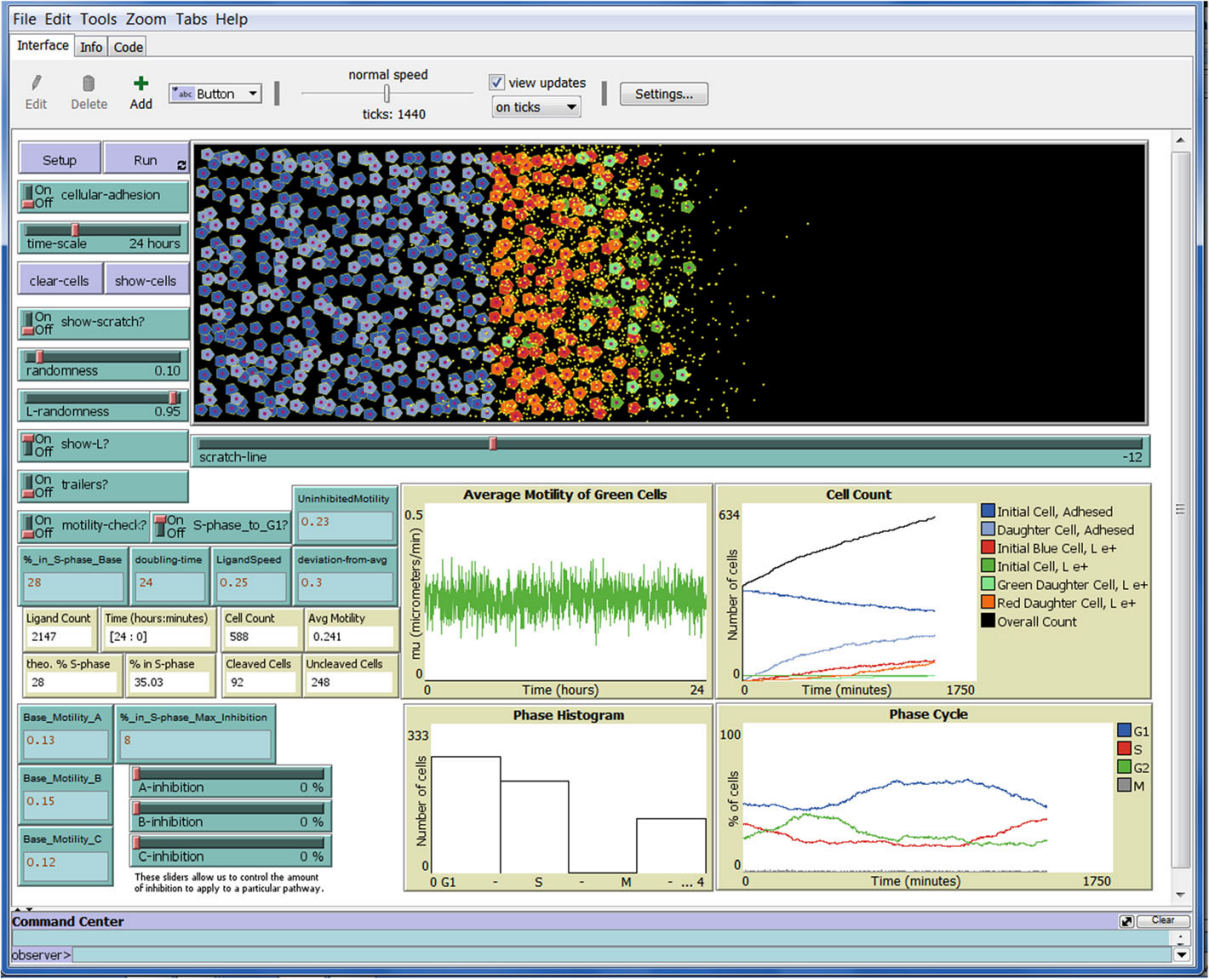
Cells at end of 24 h simulation. Shown is the same simulation set up in Fig. 2 after running for 24 h. Cells at the original scratch edge (green) have migrated into the open space along with their daughter cells (light green), blue cells that have crossed the scratch line (red), and their daughter cells (orange). Cells that have passed the scratch line release L1ecto (yellow dots). Outputs are shown in the tan boxes and graphs

The remaining figures show example simulations in several environments at the end of the simulations. Figure 3 shows the endpoint of a 24 h simulation of the setup shown in Fig. 2, showing cells and ligand. Figue 4 shows the endpoint of another simulation run under the same settings, but without showing ligand or cells. “Show-L?” was turned off and cell “trailers” were turned on before the simulation was run, and the “clear-cells” function was engaged after the simulation ended. This allows the paths taken by the initial green cells to be seen clearly as different colored trails without being obscured by the numerous daughter cells or ligand. One will notice that turning on the cell trailers slows down the simulation as it proceeds to accommodate the increased tasks. Note the farthest distance to the right that each individual cell travels is highly variable, which creates a very uneven edge at the end of the simulation. This is an accurate reflection of cell behavior that we have observed in our *SuperScratch* assays, which makes conventional scratch assay measurements by drawing straight lines very inaccurate, as we pointed out in Fotos et al. [5].

**Fig. 4:**
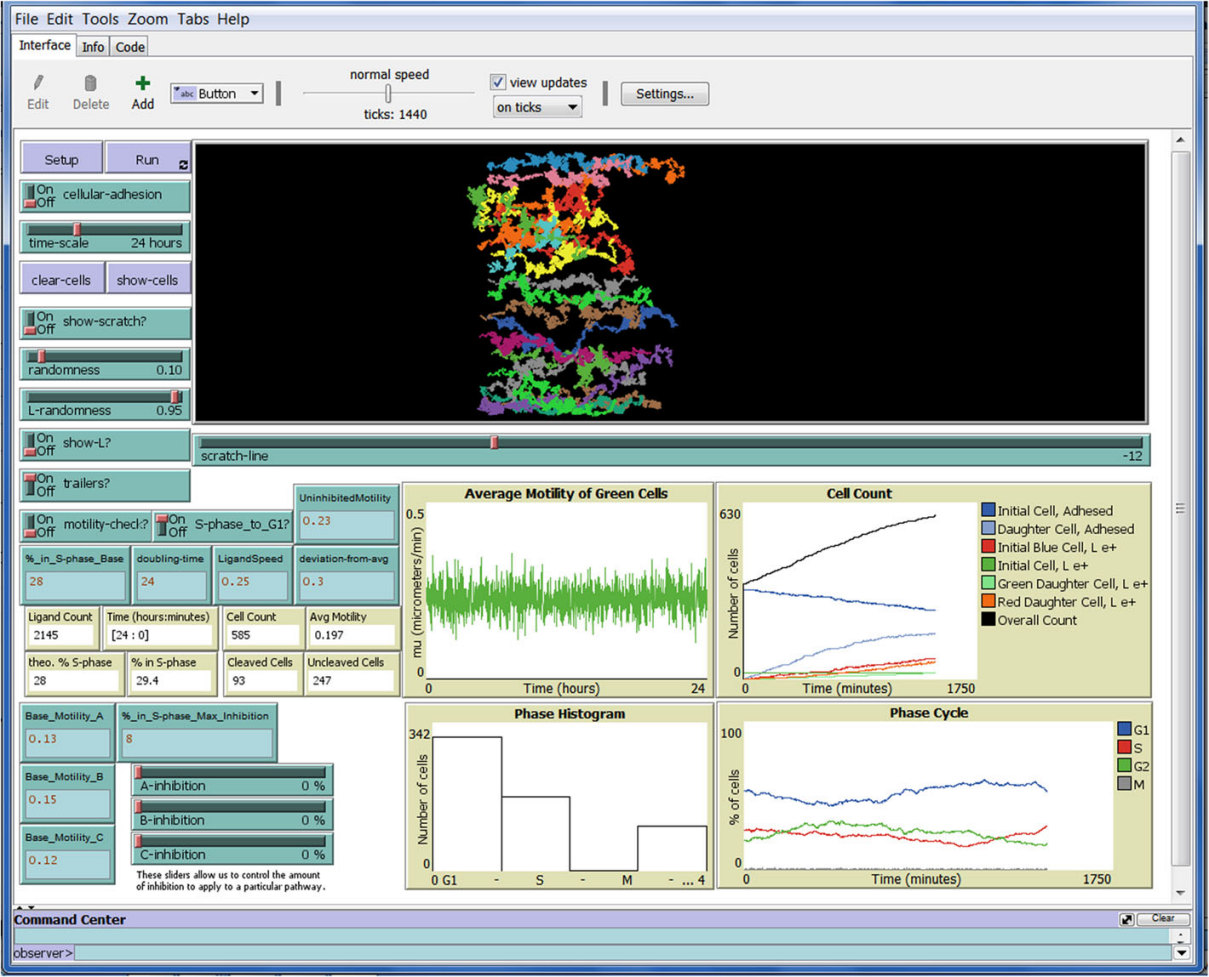
End of 24 h simulation with cell trails but without cells. Shown is another simulation under the same settings as in Fig. 3, but with “trailers” turned on and “show-L?” turned off before the simulation was run. After the simulation was run, “clear-cells” was engaged to remove display of all cells. This results in clear visualization of the trails of the original green cells at the initial scratch line

**Fig. 5:**
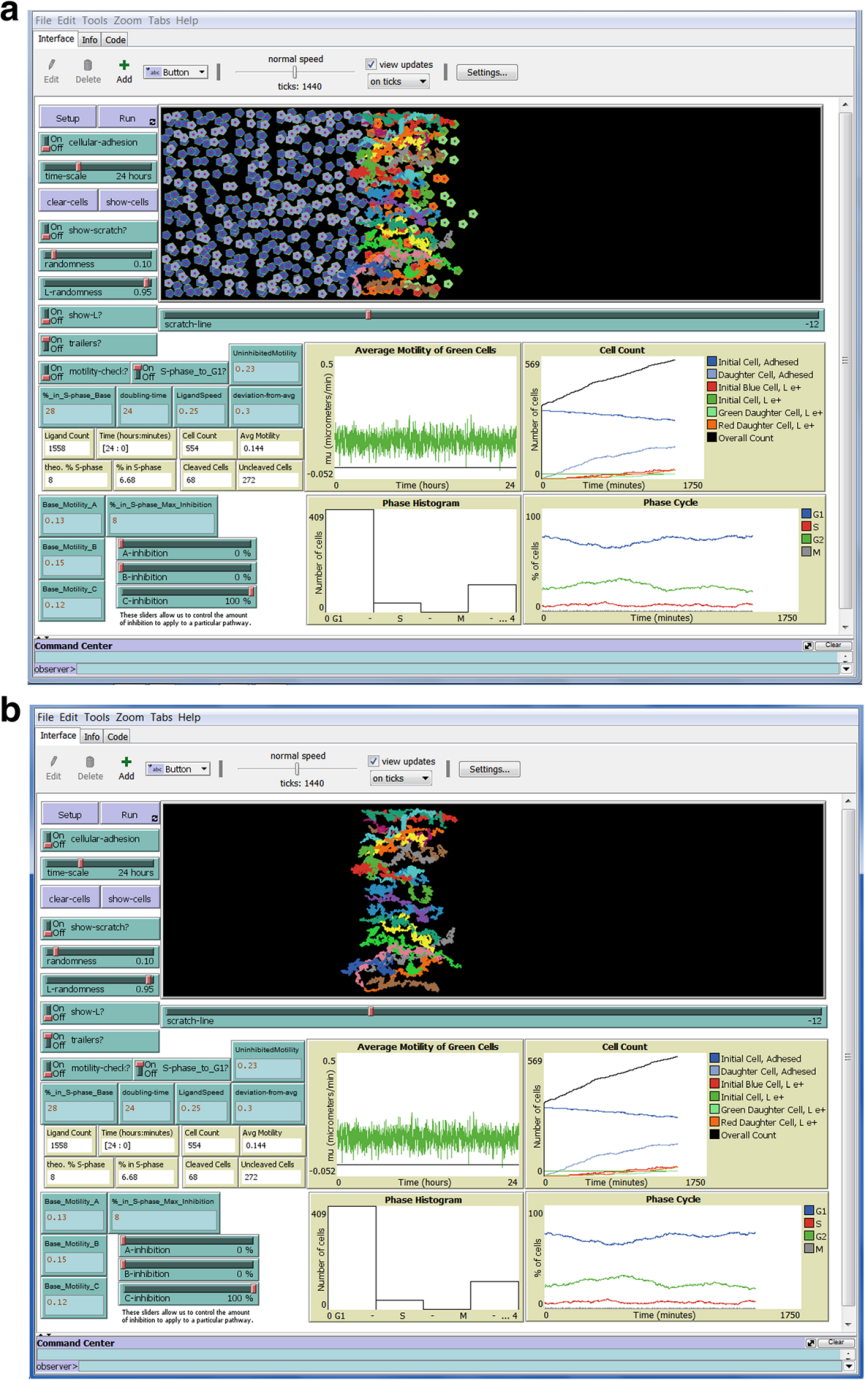
Cells at end of 24 h simulation with C pathway fully inhibited. A simulation was run under the same conditions as in Figs. 3 and 4, but with pathway C fully inhibited by engaging the “C-inhibition” slider all the way to the right (100%). a. “trailers?” were turned on, and “show-L?” was turned off before the simulation was run. b. “clear-cells” was engaged to leave only the cells trails. Cell migration was clearly reduced, as evident by the shorter cell trails (compare to uninhibited cell trails in Fig. 4)

Figure 5a shows the endpoint of a simulation with intracellular signaling pathway C (FAK) fully inhibited. Shown are the cells along with their pathway trailers. The differences in the trail lengths can be seen more clearly in Fig. 5b without the cells shown. Note that cells have migrated less far to the right than in Figs 3 and 4 due to inhibition. Also note that proliferation is decreased, and that a few values on the Average Motility graph went negative, which caused the zero line to move up on the Y-axis. Also note in Fig. 5a that several cells without trails are at the far right leading edge (i.e., the tracked cells at the initial scratch edge are not always those that move the farthest to the right). This is because each cell makes motility decisions based on rules that are independent of the decisions of other cells. Here, a few light green daughter cells (no trails) happened to migrate farther than any of the dark green cells (with trails) being tracked. This is an accurate reflection of cell behavior that we have observed in our *SuperScratch* assays.

**Fig. 6:**
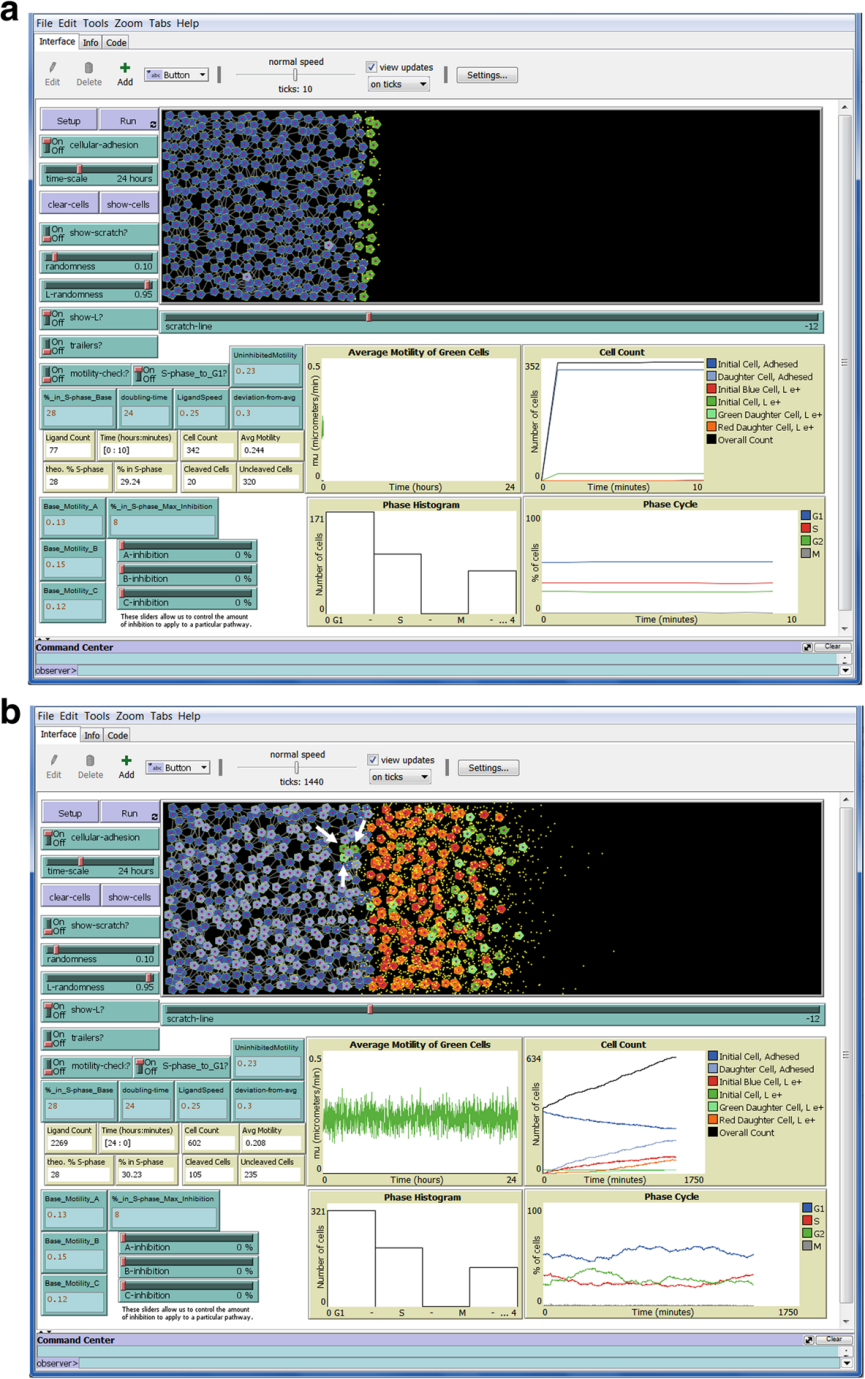
Simulation with cellular adhesion on. “cellular-adhesion” and “show-L?” were turned on before the simulation was run. a. Simulation was stopped after only 10 min had elapsed on the Time indicator box. Note the intercellular connections (lines) between the blue cells behind the scratch line. Green cells are not bound by adhesive bonds and are beginning to release L1ecto (yellow dots). b. At the end of the simulation, many cells have migrated beyond the scratch line into open space and are releasing L1ecto. Cells behind the scratch line remain interconnected by adhesive bonds. Note that 3 green cells have migrated back into the confluent monolayer (white arrows). Such cells often reestablish adhesive bonds with neighboring cells

Figure 6a shows the beginning of a simulation with the “cellular-adhesion” switch turned on, after 10 min has elapsed on the time indicator, showing cells and ligand. Note the interconnections between cells behind the scratch line, but lack of such adhesions for the green cells at the scratch edge. Also note that the graphs and tan indicator windows all show values up to that point. Figure 6b shows the endpoint of this simulation after 24 h. Running such a simulation with the cellular adhesion function engaged takes considerably longer than with adhesion off. Note that an initial green cell and two green daughter cells migrated back into the confluent monolayer (i.e., to the left; white arrows). These cells often re-establish adhesive connections with surrounding cells in the monolayer. This infrequent cell migration back into the monolayer is an accurate reflection of cell behavior that we have observed sometimes in our *SuperScratch* assays, as we illustrated in Fotos et al. [5].

**Fig. 7:**
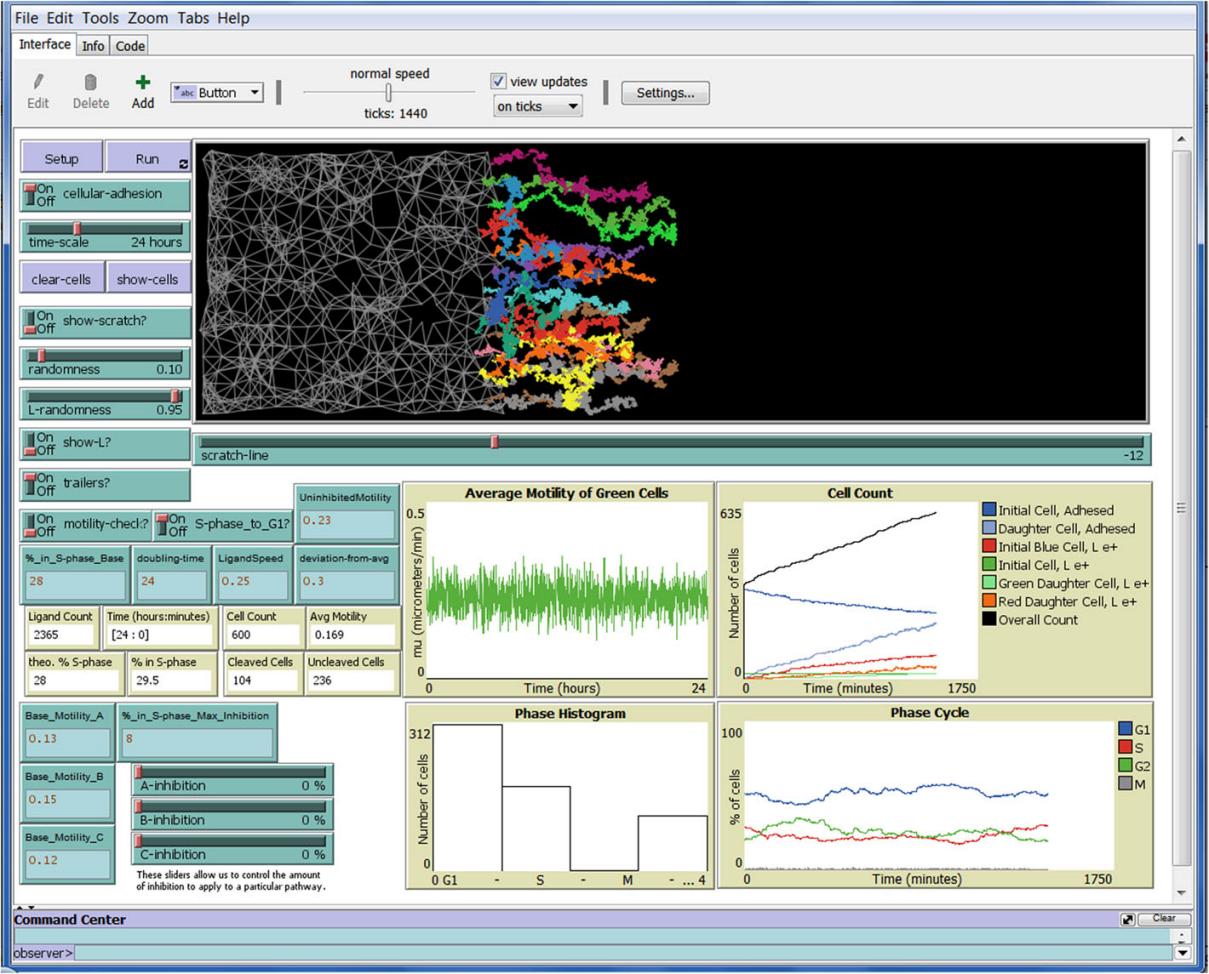
End of 24 h simulation with cellular adhesion on but without cells. Simulation was run with “cellular-adhesion” and “trailers” turned on, but “show-L” turned off. At the end of the simulation, “clear-cells” was engaged. Clearly visible are the pattern of interconnections of the cells behind the scratch line along with the trails of the migrated green cells that initially were along the scratch edge

Figure 7 shows the endpoint of a simulation with cellular-adhesion on, cell trailers on, without showing cells or ligand. Interconnections between cells in the confluent monolayer can be seen clearly, as can the trails of the cells that migrated away. This illustrates the versatility of our simulation program not only to change multiple parameters, but also to visualize those aspects on which one wishes to focus.

**Fig. 8:**
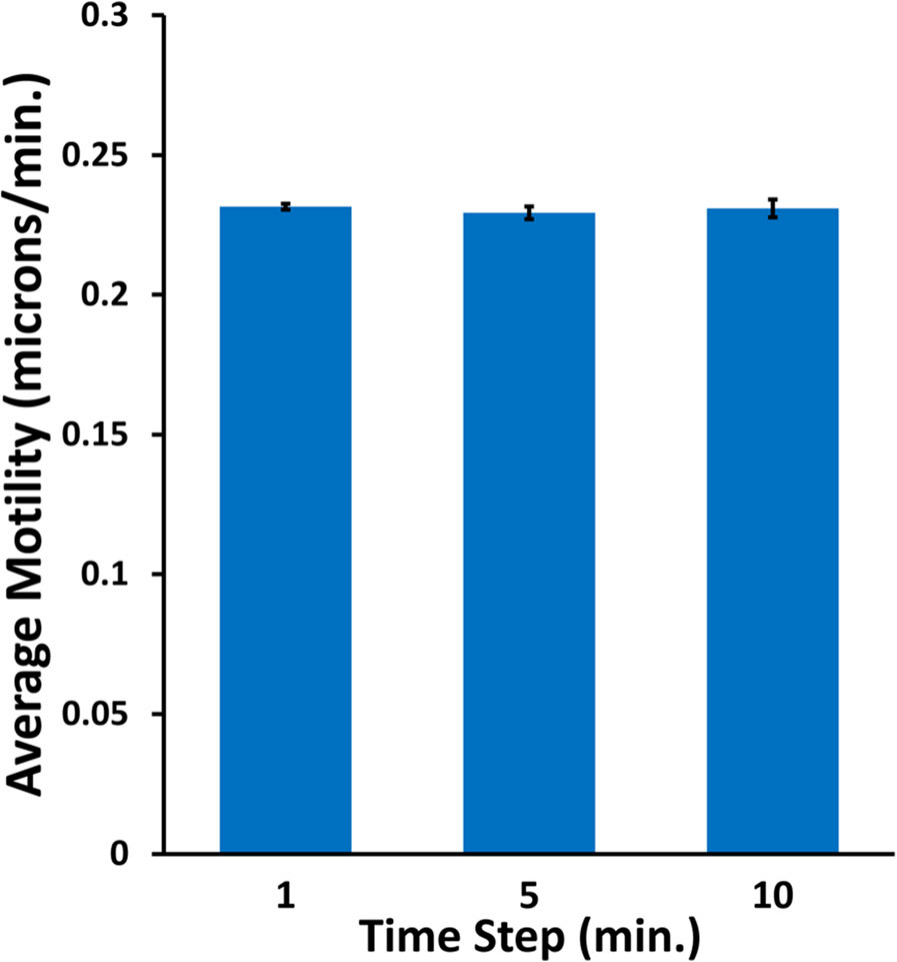
Comparative analysis of average motility at different time intervals. A simulation was run at the standard values shown in Fig. 3, data from the average velocity graph was exported into Microsoft Excel, and average velocities were calculated using values at time intervals of every 1, 5, and 10 min (ticks). Average motilities were plotted. Error bars = s.e.m.

Our simulation gives velocity data for each tracked cell every minute (tick). However, images of actual cells are acquired and tracked every 5 or 10 min using MetaMorph software in the Galileo laboratory. To ensure that the average motility (microns/min.) does not change regardless of 1, 5, or 10 min averages, the data from the average motility graph after a simulation was exported into Microsoft Excel, and averages were calculated using velocity values at time intervals of 1 min (all values), 5 min, and 10 min. As can be seen in Fig. 8, the averages using values from the three different time intervals resulted in identical averages equal to the value entered into the simulation (0.23 μm/min.). This is not the same way that tracking actual cells in MetaMorph is done, as that program calculates the velocities of the cells based on their absolute positions at the specified interval (e.g., 5 min.). NetLogo does not store the absolute positions of the cells, so our calculations at 5 and 10 min intervals could not be calculated from the positions at time 0, 5, 10, etc. as is done in MetaMorph. Nonetheless, this comparative analysis at different time intervals revealed that the averages are correct regardless of the time interval, as is found experimentally.

### Life as a blue cell

Following is a description of the environment that governs the behavior of a blue cell within our simulation. Each cell is an independent model, which is what makes this an agent-based model. On every tick, NetLogo updates each individual cell. The blue cells are those that are initialized behind the scratch line. Each is created with different initial parameters. The “A” pathway is calculated to be a random number within a range. Variability is a variable in the model that reports the “deviation-from-avg” that is manually entered. The range mentioned to calculate the speed through the A pathway is the calculated motility of A (depending on the pathway’s inhibition) plus or minus the variability. If A is uninhibited, the value of its motility is the user-defined “UninhibitedMotility”. However, if the pathway is fully inhibited, the motility of A is the user-defined “Base_Motility_A”. The cells are also randomly assigned to a phase of the cell cycle, with the phase distribution similar to that of experimental values. Since the cell is blue, it has yet to shed its L1 ectodomain, and therefore has a variable noting this.

On each tick, cells first are told to “update-params”, a function that updates the parameters of each cell. Looking at the block following the spring constant code:
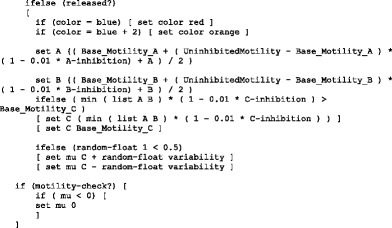


On each step, a blue cell’s A and B values have a 50 % chance of fluctuating slightly by adding or subtracting two different random numbers within the variance range. Next we increment the cell’s internal cycle, and then call “update-phase”.

“Update-phase” checks the cell’s internal cycle counter against the calculated ranges for each phase in the cell cycle. If the cell is in the M-phase, the function mitosis is called, which creates a new daughter cell if there is open space for it. This would be a periwinkle cell, to signify that it was not there at the beginning of the simulation.

Next, the blue cell calls the function “cell-diffuse”. This handles the cell’s movement. It first looks at the eight surrounding patches and checks to see if any of them are empty. Empty patches in the neighborhood are stored in the cell’s memory for this tick. Next, the cells check if the patch behind it has more than two cells. If this is the case, the cell will move forward to allow more room for the dense cells behind it.

Next, assume the variable “randomness” from the interface is set to a base value of 0.10. This signifies that there is a 10% chance that the blue cell will take a random walk in any direction at the speed mu that is just the maximum between the variables A and B with inhibition accounted for. There is a 90% chance that the cell will turn to face the surrounding area of least density and move towards open space at the same calculated motility rate.

Now that the cell has moved, it must be analyzed from the new position. In the function “L-production”, it is checked if the cell has moved past the initial-scratch line, and if there are any cells in front of it. If it is past the line, and there are less than two cells ahead of it, the cell will shed the L1 ectodomain and change from blue to red. The cell now will behave according to different rules. It will hatch 4 yellow L1 molecules into the NetLogo environment when it becomes red. If the cell moves back into the pack of cells undergoing adhesion, then it will become blue again as it will regain its adhesion properties.

## Discussion

Herein, we have developed a rule-based simulation that models the migratory and proliferative behavior of GBM cells in our commonly used 2-dimensional *SuperScratch* assay (see Additional file 1 for NetLogo file). The conventional “scratch” or “wound healing” assay is one of the most widely used assays for cell migration. Undoubtedly, this is because of its simplicity, but there are several limitations and drawbacks. That is why we developed the *SuperScratch* assay whereby the cells along the scratch edge were tracked individually and precisely in Fotos et al. [5] where we compared the two assays directly. In the conventional scratch assay, and any 2-dimensional motility assay that measures an advancing front of cells imprecisely (e.g., by drawing a line), advancement of the front is a combination of active migration and passive filling of space by newborn daughter cells. In the *SuperScratch* assay and its simulation presented here, the movement of individual cells is measured, and not the filling of a gap. Individual cells can be affected by their neighbors, but the filling in of a gap is not measured. Usually, active migration by cells originally at the scratch edge is faster than filling in space by cell division. In the 3-dimensional Transwell motility assay, cell division is also irrelevant because it measures only those cells that actively squeeze through small membrane pores to the other side in response to a chemotactic signal. Thus, that assay is fundamentally different in that cells must respond to a chemotactic signal and then squeeze through small pores, which arguably assays several other cell properties beyond simply unconstrained motility.

Mathematical models for GBM previously have been developed to describe cell proliferation and/or motility in vitro [8, 12, 13, 15] or cell and tumor behavior in vivo [7, 9, 14, 18]. However, these models either have been aimed at very specific aspects of cell behavior, have been mathematically complicated (e.g., use multiple differential equations and partial differential equations), and/or use software not readily available to the public. We sought to develop a model that 1) would accurately account for the motility behavior of GBM cells that we observe under different experimental conditions in vitro in two dimensions, 2) was based on software that was freely available to the public, 3) could be coded so that each individual cell followed several simple rules to govern their motility behavior decisions, 4) could simulate cell behavior under various conditions that were adjustable by the user, 5) had parameters that were adjustable easily through a simple graphic interface, and 6) could have the code modified easily by other investigators to suit their particular needs.

Several other previous reports that model the effects of cancer cell behavior and signaling are worth mentioning for comparison. None of these models, however, fulfill all of the abovementioned criteria. A review of GBM models by Hatzikirou et al. [6] grouped different types of GBM modeling into four categories: 1) early glioma tumor growth, 2) invasion of brain by tumor cells (simulates invasion into nonhomogeneous brain structure), 3) tumor modeling of genetic alterations and their macroscopic effects (these models are interested in what microscopic changes are required for a given macroscopic behavior), and 4) modeling therapies (mainly tumor resections effects and chemotherapy). Our model would fit into the first category. Out of the three papers compared in this category, only one was an agent-based model of GBM. The movement of the cells in the agent-based model was defined by the nutrient and toxic concentration, and the mechanical confinement of the neighboring spaces in the lattice. There was no stimulation by an external molecule, as in our model.

Tanaka et al. [14] developed a hybrid compartment-continuum-discrete (CCD) model to model tumor development in vivo in two or three dimensions. Simulations were performed using MATLAB software (MathWorks, Natick, MA). They modeled a developing tumor to have three compartments: a necrotic core, a ring of proliferating cells, and an outer ring of proliferating cells from which cells become migratory at the tumor surface. Individual characteristics of migratory cells were stored, which affects the probability that they will proliferate or migrate. The cells within the tumor mass were modeled as groups of cells. This minimized the computational requirements for the model. This hybrid model appears to accurately simulate in vivo growth of a GBM tumor and general motility and invasive cells. Our model solely simulates a two-dimensional cell monolayer situation, but allows for easy manipulation of signaling pathways that affect motility and proliferation through the user interface.

A model by Swanson [13] is based off of Chicoine and Silbergeld’s [2] two dimensional radial dish assay experiments. It is a diffusion based model where their goal was to look at the motility and proliferation of the GBM cells. Consequently, they “poisoned” the cells to prevent mitosis, but it is assumed that the “poison” does not affect the motility of the cells. They ultimately measured the density of cells vs. the radial distance from the center at different time intervals. The results of Chicoine and Silbergeld suggest a minimum linear velocity of 4.8 μm/h in vivo. The mathematical model is simply (∂c/∂t = ∇ (D(x)∇c) + ρc), which is the sum of the diffusion of the cancerous cells and the net proliferation of the cancerous cells. This model does not, however, have a graphic user interface or allow for easy changes to cell conditions.

An in vivo 3-D model of brain cancer was developed by [18] to simulate glioma cell motility, proliferation, quiescence, and apoptosis. A four quadrant model of a brain slice was fed glucose and transforming growth factor alpha and monitored for volumetric tumor growth, tumor heterogeneity, expansion rate per tumor region, expansion rate per clone, phenotypic spectrum per tumor clone, and molecular phenotype switching profiles. Chemotaxis was modeled using a diffusion based system of differential equations, but has a parameter that allows for stochastic movement. The glioma cells mutate through a linear progression throughout the simulation, with different sensitivities to the environment based on the cell’s progression towards a new clone. Cells are modeled individually and have equations governing all aspects of cell life, but stochastic parameters are incorporated based on random probability. This model was implemented in Java, combined with in-house developed classes for representing molecules, reactions and multi-receptors as a set of hierarchical objects. The code appears to be unavailable.

Kim et al. [9] developed a complicated multi-scale model that models a population of glioma cells that either proliferate or migrate depending on the availability of glucose. They used a system of three ODEs (ordinary differential equations) for the miR-451-AMPK-mTOR intracellular signaling pathway, which is dependent on glucose levels. The molecule levels or activation of each of these species in a cell determines whether it will be in the proliferative state or in the migratory state. The ODEs utilized three interconnected ODEs, only the first of which was directly dependent on an extracellular molecule (glucose). They used a lattice-free cell-based model which models each cell as an ellipsoid that applies and is subject to forces from other cells and the extracellular matrix. In order for a cell to move, it must apply a force to its surroundings. They used a PDE (partial differential equation) model for the distribution of extracellular molecules such as oxygen, glucose, chemoattractants, extracellular matrix (ECM), matrix metalloproteases, and chemotherapeutics. The code for this model does not appear to be available.

Based on these and other GBM models found, there has not been a published paper that uses L1 stimulation of GBM cells or the NetLogo computational platform. Our modeling platform is unique in its simplicity, accuracy, ease of use, and ability to be adapted by others. NetLogo can operate via rule-based code as well as mathematical code and so they are both incorporated in this model (see NetLogo tutorials for specifics). Sensitivity analysis can be performed fairly easily by changing parameters and observing significant deviations from base behavior. It is possible to write code to run multiple simulations rather than look at each case interactively with slider bars. Although actual experiments can be conducted to find parameters, one can use qualitative feedback to improve on parameter guesses.

Our model of GBM cell motility and proliferation is fundamentally different from those above because it is based on a set of simple rules and probabilities that determine the behavior of the agents (i.e., cells and L1). In doing this, simulations run with a particular set of inputs are very similar in their overall outcome, yet each simulation is unique because the behavior of individual cells is independently determined at each time point (tick). Although this stochasticity might be viewed as an inaccuracy when compared to a model based on equations, this characteristic makes the modeling platform more representative of biological systems, where stochasticity is a significant component. We believe that the presented set of hierarchical rules reflects, and is sufficient to explain, the GBM cell behavior that we have observed in *SuperScratch* assays in the laboratory. These simulations also likely will make predictions of cell behavior that we have yet to test experimentally, which might allow estimates to be drawn before conducting experiments. The main drawback of our model is that it is limited to a two-dimensional cell culture paradigm. However, this arguably is also its strength for those interested in modeling cell motility and proliferation in a dish.

Specifically, our initial modeling behavior is determined by basing the rules primarily on one cell type (T98G). However, rules in the code can vary with different cell types. A simple example would be to uncouple proliferation from motility in the code, as pointed out above, to allow proliferative foci potentially to form if insufficient space for migration occurs in specific areas and not others. Another example would be to have two different adjacent cell areas, where one has cells releasing a ligand (e.g., L1ecto) and the other has cells not releasing it, but responding to the gradient from the nearby releasing cells (paracrine). The paracrine responding cells could be set up to use different rules and/or equations from the autocrine stimulated releasing cells. One could even create subpopulations within each of the two populations, thus creating a very complex model of cell migration and proliferation, which could generate behaviors that could be tested experimentally in the laboratory. We have begun to develop such modifications of our framework but are only at the beginning stages.

## Conclusions

We developed a simple rule-based model that appears to accurately depict glioblastoma cell behavior during *SuperScratch* assay experiments performed in the laboratory. It is tempting, therefore, to speculate that actual observed cell behavior in a dish might be governed by a similar, if not the same, set of rules. We realize that it is not known whether or not the computational behavior in our simulations actually reflects correct implementation of biological processes. Proving that, one way or the other, is well beyond the scope of this work, and likely is not even determinable with the current knowledge of how cells work. That our model has built-in stochasticity is a strength in that this reflects biological systems. How one might move forward could be to alter the number and connectivity of critical variables, to alter assumptions and the rules based on them, and to change various parameters. This could be done both to develop simulations that better fit the observed behavior of other cell types, and to make predictions of how alterations of variables or parameters will result in changes in observed cell behavior.

By utilizing NetLogo, this model is freely available for interested investigators to examine, use, and modify to suit their own individual purposes. The simplicity behind the NetLogo interface is attractive, as it makes alterations by others a relatively easy task. Information about how the model works is explained herein and also can be found under the “Information” tab of the model. This model should be useful to researchers of cell motility in vitro and to instructors and students of cell biology covering cell motility. It is hoped that our model and simulations can be used in cell biology teaching labs by students alongside their performing commonly used cell culture scratch assays.

## Additional file

Additional file 1: NetLogo file of the modeling framework described in this article that simulates autocrine/paracrine stimulation of glioblastoma cell motility and proliferation by L1CAM in 2-D culture. The NetLogo software can be downloaded at https://ccl.northwestern.edu/netlogo/. (NLOGO 28 kb)

## Abbreviations

ECM: Extracellular matrix
FAK: Focal adhesion kinase
FGFR: Fibroblast growth factor
GBM: Glioblastoma multiforme
L1: L1CAM adhesion/recognition protein
ODE: Ordinary differential equation
PDE: Partial differential equation
